# Motivational Determinants of Objective Physical Activity in Women with Fibromyalgia Who Attended Rehabilitation Settings

**DOI:** 10.3390/jcm10235547

**Published:** 2021-11-26

**Authors:** María-Ángeles Pastor-Mira, Sofía López-Roig, Fermín Martínez-Zaragoza, Eva Toribio, Ainara Nardi-Rodríguez, Cecilia Peñacoba

**Affiliations:** 1 Department of Behavioral Sciences and Health, Miguel Hernández University, 03550 Alicante, Spain; mapastor@umh.es (M.-Á.P.-M.); f.martinez@umh.es (F.M.-Z.); anardi@umh.es (A.N.-R.); 2 Fibromyalgia Unit, Hospital of San Vicente del Raspeig, 03690 Alicante, Spain; toribio_eva@gva.es; 3 Department of Psychology, Rey Juan Carlos University, 28922 Madrid, Spain; cecilia.penacoba@urjc.es

**Keywords:** biopsychosocial, chronic pain, fibromyalgia, physical activity self-efficacy, goal preferences, activity avoidance, physical activity behavior, rehabilitation

## Abstract

Being physically active has positive effects on fibromyalgia functioning. However, promoting an active lifestyle in these patients continues to be a relevant clinical challenge. Our aim was to test a motivational model to explain light (LPA) and moderate-vigorous physical activity (MVPA). A cross-sectional prospective study was conducted at a tertiary level of care. Participants completed sociodemographic, clinical, motivational (physical activity self-efficacy and goal preferences) and behavioral measures (activity avoidance). LPA and MVPA were measured with triaxial accelerometers, starting the same day of the aforementioned assessment. Out of 211 women, 183 completed this measure. Structural models were performed. Our results show that the best fit indices (CFI = 0.97, SRMR = 0.04) showed a model with direct influence of PA self-efficacy on MVPA (*p* < 0.01) and indirect influence on LPA (*p* < 0.001). LPA received the influence of PA self-efficacy mainly through activity avoidance (*p* < 0.01). Clinical variables did not have any effect on PA intensities. Thus, the motivational variables showed different paths to explain two PA intensities. Targeting PA self-efficacy in rehabilitation settings is needed to enhance both daily LPA and MVPA intensities.

## 1. Introduction

Pain is an aversive stimulus that triggers avoidance and inactivity responses and may become an obstacle in being able to reach personal goals [1]. Patients with chronic pain usually have a sedentary lifestyle which leads to impaired fitness which, in turn, decreases their physical function and increases the risk of suffering comorbidities [2]. Increasing physical activity is a relevant non-pharmacological rehabilitative strategy in order to improve their psychological and physical health status and to enhance their functional autonomy [3]. The current motivational perspective of chronic pain states that people with this problem are faced with multiple goals which are often incompatible with each other [4,5,6,7]. Hence, they have to prioritize choosing between pain avoidance goals and achievement goals, such as being physically active. This prioritization may well be a crucial point since it could contribute to explaining the patients’ sedentary behaviors and low physical activity.

Persistent and outstanding pain demands constant striving for self-regulation, and the preference for pain avoidance goals can often displace other goals related to enacting or maintaining physical activity, which aims to improve functioning. When the goal of controlling or relieving pain is a condition in order to achieve other personal goals, this leads to strengthening avoidance behaviors which in turn results in an increase in suffering and disability [5,8].

Fibromyalgia is a widespread musculoskeletal chronic pain condition which is associated with other symptoms such as fatigue, non-refreshed sleep, decreased attention, memory problems, anxiety and depression [9,10]. It is a potentially disabling problem and often shows a high socio-sanitary burden [11]. Fibromyalgia is more prevalent in women, and patients with this problem report high functional, emotional and daily life impact as well as less physical activity than others [12], a high proportion of sedentary behavior [13,14] and impaired subjective and objective physical function [15]. Nowadays, fibromyalgia is an important clinical challenge, with a diagnosis that remains controversial and where the best treatment options include graded physical, pharmacological and psychological strategies depending on the severity of the fibromyalgia condition [10,16]. In this health problem, one rehabilitative main target is to maintain or increase physical activity and exercise to improve physical function and avoid disability [15]. In fact, in patients with fibromyalgia, physical activity has shown positive effects on health outcomes [14,17,18,19,20,21,22,23,24]. The evidence suggests that it is better for patients to keep themselves active than to rest or stay seated, this being a well-documented therapeutic strategy to increase both physical activity and exercise [14,25]. Moreover, it is well-known that poor adherence to physical activity and exercise can limit the effectiveness of long-term health benefits [26]. However, interventions targeting physical activity have had limited success and physical activity promotion also remains a clinical challenge [22].

In women with fibromyalgia, preference for pain avoidance goals against different task-achievement goals has shown significant effects on more disability and fibromyalgia impact, always with activity avoidance mediation [27]. Avoidance behavior has been established as a predictor of the worst self-reported fibromyalgia physical and psychological functioning [28,29]. Despite the growing research about physical activity in fibromyalgia, to the best of our knowledge, there are no studies about the role of goal preferences and activity avoidance on objective daily physical activity. The preference for pain avoidance goals against achievement goals, such as being active, may become a self-regulatory problem for patients and undermine striving for goals related to physical activity and exercise, increasing avoidance behavior. Most studies have worked with avoidance behavior as main predictor of chronic pain health outcomes but not with objective physical activity. According to Meulders [5], the role of avoidance is fundamental in explaining the pathway to disability, and preventing said disability is an important clinical target for fibromyalgia and chronic pain patients. Clinical improvement in these patients, through maintaining constant levels of physical activity and exercise, does not only depend on behavioral advice from health personnel [30], but also on specific motivational strategies which are necessary in order to arrive at behavioral change and persistence in activity. Evidence about motivational factors lets us better understand different strategies that could be effective in increasing change. Health personnel could consider this evidence in their behavioral prescriptions, once we have a deep understanding about how these factors influence physical activity performance.

From the social cognitive theory [31,32], there is evidence about the association of goals and self-efficacy with physical activity [33]. Taking into account a self-regulation point of view, self-efficacy is a key factor as a strong motivational determinant of behavior. Self-efficacy affects the degree to which a goal is perceived as feasible and thus may influence the patients’ goal preferences. There is evidence about the role of self-efficacy on physical activity and exercise in both clinical and non-clinical contexts [34,35,36]. Moreover, self-efficacy is the main factor in affecting psychological and physical functioning in chronic pain patients [37,38] and predicting exercise and physical activity in fibromyalgia [39,40].

**Figure 1:**
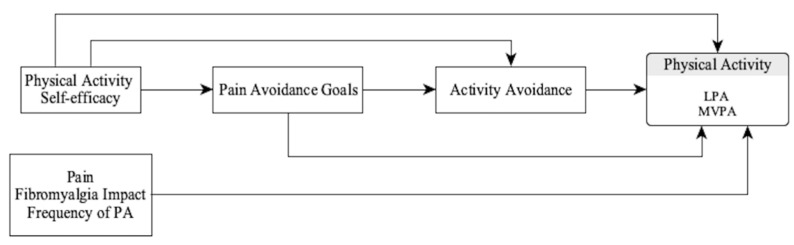
Initial motivational model. PA: Physical activity; LPA: Light physical activity; MVPA: Moderate-Vigorous physical activity.

Therefore, in women with fibromyalgia, this study aimed to test a motivational encompassing model of objective physical activity intensities (Figure 1), taking into account well-established variables such as self-efficacy, and testing its influence on physical activity through the mediation of goal preferences and the avoidance behavioral pattern, controlling the effect of clinical variables. If the results support the importance of these motivational factors, we will have well-known resources in rehabilitative settings, such as self-efficacy and goal management, to support self-regulation efforts in increasing physical activity.

## 2. Materials and Methods

### 2.1. Participants

The participants were 211 women diagnosed with fibromyalgia who attended the Fibromyalgia Unit (FU) of the San Vicente del Raspeig Hospital (tertiary level of health care). They were diagnosed following the American Rheumatology Association (ARA) criteria [41,42]. Most of them were married or living as part of a couple (67.3%; *n* = 142), with primary (47.4%; *n* = 100), secondary (33.2%; *n* = 70) and university studies (10.4%; *n* = 22). At the time of the study, 21.8% were on sick leave (*n* = 46), 6.2% were retired (*n* = 13) and 7.1% (*n* = 15) had retired due to pain. Only 25.6 % of women were working out of the home (*n* = 54) and 19% were housewives (*n* = 40). The mean age was 52.6 (SD = 8.0) and the mean time from the first symptoms was 13.7 years (SD = 9.3) and 7 years (SD = 7.3) from the diagnosis of fibromyalgia. Out of 10, the mean of pain intensity perception was 7 (SD = 1.6).

### 2.2. Variables and Instruments

Socio-demographic and clinical variables were measured with “ad hoc” questions.

Physical activity self-efficacy: We used the total score of the “self-efficacy for physical activity scale” (SEPAS) [43] to assess the confidence people felt in doing regular physical activity and exercise, despite several barriers identified in a previous study with women with fibromyalgia: pain, fatigue, bad weather, feeling stressed, sad and worried and having a bad day due to fibromyalgia [44]. The SEPAS comprises 25 items answered on an 11-point scale (0 = not at all confident, 10 = completely confident) and assesses the self-efficacy for brisk walking in both 30 and 60-minute sessions (Factor I), for performing daily physical activities (Factor II) and for undertaking moderate physical activity (Factor III). Higher scores indicate higher self-efficacy. The internal consistency in this sample for the entire scale was α = 0.97.

Pain avoidance relative to physical activity goal preferences: We adapted the Spanish version [27] of the goal pursuit questionnaire (GPQ) [45] to consider physical activity and exercise as an achievement goal in physical activity tasks (GPQ-PA). The GPQ assesses the habitual pursuit of goals in people who experience pain, taking into account short-term goals relative to achievement or long-term goals, which can be activated at the same time in one specific situation. Following the same GPQ design and wording, the GPQ-PA comprises five items which, with a vignette format, present a situation related to physical activity and exercise. Participants rated their preference for maintaining the activity (achievement long-term goal) or avoiding pain (avoidance short-term goal). The situations were based on (1) walking while taking advantage of other daily activities (such as going to work, shopping or taking the dog out), (2) brisk walking for exercising 30 or 60 min, which is the minimum and the standard walking exercise recommendation for women with fibromyalgia in our research [46], (3) the three levels of physical activity (light, moderate and vigorous) usually assessed in physical activity questionnaires [47], and (4) the fibromyalgia widespread pain. Participants were to imagine themselves, as vividly as possible, in the presented situation (i.e., “While you are walking, taking advantage of going to work, shopping or taking the dog out, your body becomes increasingly painful. You are expected to complete your walking route today”). Each vignette is followed by a sentence showing a thought which indicates a goal preference (i.e., “I think it is more important for the pain in my body to be reduced now than finishing my walking route") that participants had to rate on a 6-point Likert scale (1 = strongly disagree, 6 = strongly agree). Higher mean scores indicate stronger preferences for short-term hedonic goals (avoiding pain) relative to achievement goals (maintaining the activity). Due to it being a novel scale focused on physical activity and exercise tasks, a preliminary psychometric analysis is presented in the result section.

Activity Avoidance pattern: We used the total score of the corresponding subscale of the activity pattern scale (APS) [48], which comprises eight three-item factors and has showed good psychometric properties. From a multidimensional point of view, the APS assesses the avoidance, persistence and pacing patterns of chronic pain patients. Items are answered on a five-point Likert scale from 0 (never) to 5 (always). The internal structure of avoidance and persistence have been reproduced in women with fibromyalgia [49]. In this study we only used the activity avoidance factor due to the high chronicity of the sample and our previous results, which have shown the relevance of this activity pattern in fibromyalgia functioning [27]. The activity avoidance pattern assesses the avoidance behavior due to the patients’ own chronic pain condition (“I have not been able to carry on with my usual level of activity”; “because of my pain most days I spend more time resting than doing activities”; “I have to put parts of my life on hold”) and not the avoidance behavior related to perceived or anticipated pain fluctuations (pain avoidance pattern: i.e. “I stop what I am doing when my pain starts to get worse”). The Cronbach’s alpha of the activity avoidance subscale in this sample was 0.66.

Pain intensity: With four items answered on an 11-point numerical rating scale (0 = “no pain at all” and 10 = “the worst pain you could imagine”) we asked for the maximum, minimum and usual pain intensity during the last week and pain intensity at time of the assessment. High mean scores indicate high pain intensity. The internal consistency in this sample was α = 0.80.

Fibromyalgia impact: Measured by the total score of the Spanish adaptation of the FIQ-R [50]. Items are answered on an 11-point numerical rating scale with different verbal anchors, depending on the item. The FIQ-R assesses both physical (fatigue, pain or muscular stiffness among others) and psychological symptoms (anxiety, depression) of fibromyalgia and their interference in daily living tasks and in quality of life. Higher scores represent higher fibromyalgia impact perception (α = 0.89).

Frequency of physical activity: We used the mean score of five “ad hoc” items to obtain the frequency of the physical activity variable. Patients were asked how many times in the seven days previous to the assessment did they do at least 30 min of daily walking, 30 min of light and moderate physical activity and 20 min of vigorous activity (i.e.: “In the last seven days, how many times did you walk at least 30 min while taking advantage of going to work, shopping or taking the dog out?”).

Outcome variables: Physical activity (PA). Objective PA was recorded with ActiGraph (Pensacola, USA) GT3X-BT accelerometer (see procedure section for details). Light, moderate and vigorous PA intensities were expressed as minutes per day and calculated taking into account the following PA vector magnitude cut-points [14,51]: 200–2689, 2690–6166 and ≥6167, respectively. For this study, we used the average time of all valid days of light (LPA) and moderate-vigorous PA (MVPA, as the sum of moderate and vigorous PA).

### 2.3. Design and Procedure

This work corresponds to the second study of a broader research, which aims to identify a physical activity self-regulation model in women with fibromyalgia in rehabilitation settings. Inclusion criteria were: women, aged between 18 and 70 years, with fibromyalgia diagnosis confirmed by the FU, following ARA criteria [42], with the ability to properly fill out the self-reported measures and committed to wearing an accelerometer for 9 consecutive days. A convenient sample of 245 consecutive new FU patients who met the inclusion criteria were invited to participate in the study. Two hundred and eleven (85.1%) accepted and signed the informed consent. There is no exact formula to calculate sample size for the structural equation modelling (SEM) statistical approach, but simulation studies [52] show that a reasonable sample size for conducting SEM is more than 150 subjects. The assessment was performed before starting any medical, occupational, physical or psychological treatment at the FU.

We conducted a cross-sectional prospective design with two measurement times where accelerometer variables were recorded for 9 consecutive days starting from the day of the first assessment. Activity counts were measured at 30 Hz and stored at an epoch length of 60 s. Non-wearing times were established in an interval of 60 consecutive, with counts = 0, and were excluded from the analysis. Participants wore the accelerometer around the hip, under clothing, fastened with an elastic belt. Women had to take it off for showering, swimming, and sleeping at night. All participants were instructed to wear the accelerometer, took a sheet with all the information and were given a telephone contact number in case of any problem. They signed a commitment to return the device within the stipulated time. Data download, cleaning and analysis were performed with ActiGraph software (ActiLife version 6.13.3). The first and last day were not excluded, assuming the same reactivity bias for all participants. Data was included in the analysis when participants had at least 10 hours of wear time (valid day) on any five days of the recording period, with at least one weekend day [51]. We obtained 26 invalid cases that did not reach the minimum amount required of valid days and two participants did not wear the accelerometer (calendar days = 0). Thus, the statistical analysis for testing models was conducted with 183 women.

### 2.4. Statistics

The SPSS v25 and the R Statistical Package [53] were used for descriptive and psychometric analysis. A principal component analysis was conducted to explore the GPQ-PA internal structure. Cronbach’ alpha was used to test the scale internal consistency.

A serial multiple mediation was tested. The analysis of model fitting was conducted with a structural equation modelling approach (SEM) by lavaan package in R [54]. The results were reported following the recommendations given in the classical study by Raykov and colleagues [55]. Based on the raw data, the correlations (Table 1, see in results section) were converted to a covariance matrix to be used with the mentioned software. SEM is based on the assumption of normality of scores. Mardia’s multivariate normality test and Shapiro–Wilk univariate normality tests were calculated using the MVN package in R [56] and showed non-normal data distribution. Therefore, a maximum likelihood estimation with robust standard errors and a Satorra-Bentler scaled test statistic was used.

A fit criteria assessment was conducted according to the Hu and Bentler study [57]. The goodness-of-fit statistical test assesses the magnitude of unexplained variance; a ratio of χ2/gL <2 suggests an acceptable fit. A root mean square error of approximation index (RMSEA) below 0.06 suggests a well-fitting model. A comparative fit index (CFI) above 0.95 indicates a good fit. A standardized root mean square residual (SRMR) of less than 0.09 also indicates a good fit. The chi-square statistic provides a conventional measure of model fit. However, because of its sensitivity to sample size, two additional fit indices were used to supplement the chi-square statistic. The choice of these two indices was based on Hu and Bentler’s recommendation of a two-index presentation strategy, which was found to provide an optimal balance between Type I and Type II error rates [57].

## 3. Results

### 3.1. GPQ-PA preliminary Analysis

The principal component analysis showed one component (all five items loading above 0.80) explaining 77.22% of the total variance. Previously, KMO = 0.82 and Bartlett test = 930.83 (*p* ≤ 0.001) indicated sample and data adequacy to perform the analysis. The GPQ-PA internal consistency in this sample was α = 0.92.

### 3.2. Correlations of LPA and MVPA with Fibromyalgia Impact, Pain and Frequency of Physical Activity

Fibromyalgia impact was significantly correlated with the MVPA (r = −0.17, *p* ≤ 0.05) (Table 1). Therefore, this variable was the only one taken into account as covariate in the MVPA relationships.

### 3.3. Initial Model and Model Fit

From the entire initial model (Figure 1) we follow an improvement model strategy, where the non-significant relationships were deleted and tested the fit of the next model. In order to economize the result presentation, we only show the intermediate and final models.

**Figure 2:**
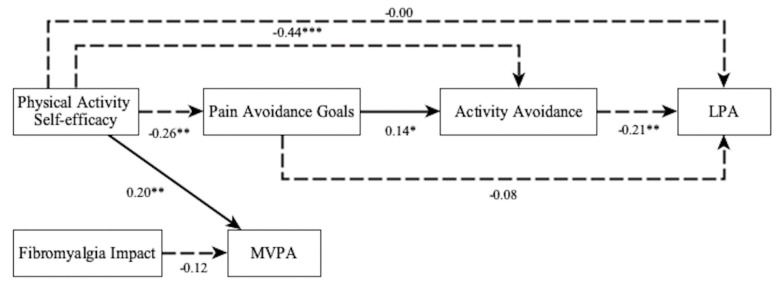
First fitted model. LPA: Light physical activity; MVPA: Moderate-Vigorous physical activity. * *p* < 0.05, ** *p* < 0.01, *** *p* < 0.001

First, the fit of the following model was evaluated (Figure 2): PA self-efficacy as an antecedent and fibromyalgia impact as a covariant. PA self-efficacy influences, directly and indirectly, activity avoidance pattern by means of goal preferences. Goal preferences influence, directly and indirectly, by the mediated effect of activity avoidance, the average of LPA and MVPA. PA self-efficacy directly influences LPA and MVPA. Fibromyalgia impact influences the MVPA.

Figure 2 represents the results of the tested model, with exogenous and endogenous variables. The observed variables are presented inside a box. The arrows indicate the directionality of the relationships among the variables. Dashed lines indicate negative relationships. The represented model includes six observed variables. All variables were measured on an interval rating scale. Standardized values are represented.

**Figure 3:**
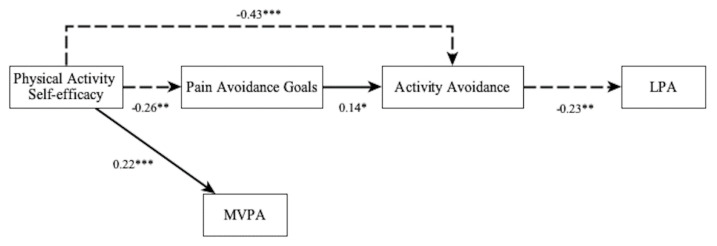
Final fitted model. LPA: Light physical activity; MVPA: Moderate-Vigorous physical activity. * *p* < 0.05, ** *p* < 0.01, *** *p* < 0.001

The final model being the same as the previous one but subtracting the fibromyalgia impact influence over MVPA and the mediational effect of goal preferences and activity avoidance on this variable. This represented model includes five observed variables (Figure 3).

Table 2 shows fit information for the two fitted models. First model shows five parameter estimates to be significant and three non-significant, and presents a bad fit. Final model shows six parameter estimates to be significant and one marginally significant, and presents a good fit. The comparison between the two fitted models showed significant differences between them (χ^2^diff = 11.950 (1), *p* = 0.000; BIC first model = 2053.79 (13), BIC final model = 2042.04 (10) showing slightly better the final model.

The final model advocates that PA self-efficacy influences, with a negative sign, directly and indirectly (mediated, although marginally, by pain avoidance goals: *p* = 0.08) activity avoidance pattern, which influence with a negative sign on the LPA. A significant total effect (*p* ≤ 0.001) on the LPA of the different indirect influences was found (Table 2). Also, PA self-efficacy influences the MVPA with a positive sign. These results evidence different paths influencing LPA or MVPA. R-squared indices vary from 0.05 (low size) to 0.24 (small-medium size) [58].

## 4. Discussion

In fibromyalgia, the health benefits of engaging in regular PA and exercise are well-established. However, trying to maintain an active lifestyle while suffering from this problem remains a clinical challenge, taking into account the high proportion of sedentary lifestyles [13] and patients’ low PA compared with healthy women [12]. To the best of our knowledge, this is the first study in women with fibromyalgia that tests an encompassing model about the motivational determinants of two intensity levels of objective PA that patients undertake in their daily life. In a long-lasting and outstanding pain sample of women with this chronic pain problem, we have explored the direct and indirect effects of specific PA motivational variables, such as PA self-efficacy and goal preferences between competing pain avoidance goal relative to keep physically active. In addition, we have considered the possible effect of clinical variables (pain intensity and fibromyalgia impact) and the previous frequency of PA. The relevance of this aim is based on the current recommendations about care in patients with chronic pain, who should receive management that addresses physical activity and takes into account psychosocial factors [59].

Our findings have shown two main paths of motivational variables and avoidance behavior depending on the two PA intensity levels that were studied. The mediation role of PA goal preference and activity avoidance behavior between PA self-efficacy and PA was only supported for LPA intensity. The total effect of these paths was significant. On the contrary, MVPA were only explained for the direct influence of PA self-efficacy. Both PA intensity levels were fully independent of pain, fibromyalgia impact and the previous frequency of PA. Hence, LPA and MVPA were not influenced by the patients’ clinical profile, keeping the motivational variables relevant. This important finding is not in line with previous studies in women with fibromyalgia that showed significant associations of pain and fibromyalgia impact with objective PA [14] or with walking exercise [60]. The long-lasting pain of our sample could explain the difference, because our participants could have “normalized” or integrated their painful status into their daily life. However, our results are coincident with a literature review that showed pain intensity was not relevant for objective physical activity in other chronic pain populations, although it was negatively related to self-reported physical activity [61].

Women who perceived self-efficacy to undertake PA, in spite of their usual illness barriers [44], showed less preference for pain avoidance goals or, in other words, more preference for achievement goals related to being active and, in turn, less activity avoidance behavior and more LPA. However, the indirect effect on LPA of this longer path was not significant, while the shorter path from PA self-efficacy through activity avoidance pattern was significant. PA self-efficacy women showed less avoidance behavior associated with their own illness condition and more LPA. Taking into account the fit indices of the model and the significant total effects on LPA, these indirect effects of PA self-efficacy would be supporting a double role, through motivational (goal preferences in the context of competing goals) and behavioral (activity avoidance) factors, although the effect is mainly through decreasing activity avoidance behavior. Hence, in rehabilitation settings, specific PA self-efficacy should be a therapeutic target even for maintaining or increasing daily LPA, and it should be considered in the initial assessment protocols. This may help both with undertaking an active lifestyle and setting goals related to becoming active and reducing activity avoidance, with positive consequences on PA. In this vein, a meta-analysis has shown the medium positive effect of goal setting interventions on increasing PA behaviors in adults [62]. Finally, the results of activity avoidance are in line with the negative role of this pattern on health outcomes [63] and extends it to behaviors that usually comprise of daily activities that do not require much effort (LPA) yet which remain necessary to avoid disability.

Unlike LPA, MVPA was only influenced by PA self-efficacy. MVPA comprises activities which increase heart rate and evoke feeling warm and slightly out of breath (moderate intensity), along with fast activities to evoke perspiration and breathlessness, usually achieved through sport or exercise (vigorous intensity) [61]. This result is in agreement with previous findings that enhance the role of self-efficacy in exercise adherence in chronic illness populations [34]. Even for these high intensity activities, PA behaviors are neither out of personal control, nor depend on pain or fibromyalgia impact. This is a main issue, with clinical implications due to the high chronicity of our sample at the tertiary level of health care. At this rehabilitative level, patients usually arrive with a long illness experience, with many unsuccessful treatments and with feelings of helplessness. Therefore, these findings underlined the relevance in undertaking a therapeutic strategy focused on increasing personal control, such as PA self-efficacy. Our results support previous ones in fibromyalgia about the role of self-efficacy in exercise using self-reported measures [39].

This study has some limitations to point out, mainly related to the sample composition and setting. The sample is comprised only of women with fibromyalgia attending a tertiary level of care, with a focus on rehabilitation treatment. Participants reported long-lasting (more than 10 years) and severe pain (mean of 7 out of 10). Hence, we should be cautious in generalizing results to other fibromyalgia samples, and future studies should replicate these findings in, for example, community samples. In addition, the marginal effects of PA self-efficacy through the goal preferences on avoidance behavior merit deeply analyzing these relationships in future research, with a more dynamic approach that complement objective PA recording with electronic diaries for assessing motivational variables.

The present study also has some strengths to underline: the objective measurement of PA in a daily context, the specificity of PA self-efficacy and behavior pattern measures, designed and tested on women with fibromyalgia; and, finally, the design and preliminary testing of a new promising scale that explores the patients’ goal preferences, which compete between trying to avoid pain versus trying to maintain a state of being active.

Taking into account the evidence about the efficacy of the theory-based interventions on PA behaviors from Gourlan 2016 [64], our study shows what the therapeutic target both for LPA and MVPA in women with long-standing fibromyalgia in rehabilitative settings should be. Luckily, the self-efficacy social-cognitive theory is a well-established theory that presents the objectives, the means and the procedures to modify behaviors and has a successful history of application in chronic pain. Bearing in mind the mentioned limitations, our findings have shown the different mechanisms through which PA self-efficacy can influence LPA and MVPA. From a clinical point of view, they offer a guide for practitioners on how to increase both light and moderate-vigorous objective physical activity. Prescription of PA can be done regardless of pain intensity and illness impact. Improving self-efficacy is a way to increase not only demanding physical activity but also daily-life activity. Future research should be conducted about how we can incorporate the different sources of self-efficacy in the usual clinical practice when practitioners give advice about carrying out physical activity.

## Figures and Tables

**Table 1:** Correlation and descriptive statistics for variables in the studied models.

	1	2	3	4	5	6	7	8
**1.** PA self-efficacy								
**2.** Pain avoidance goals	−0.26 ***							
**3.** Activity avoidance	−0.47 ***	0.25 ***						
**4.** Pain	0.02	−0.06	−0.09					
**5.** Fibromyalgia impact	−0.22 **	0.04	0.26 ***	0.56 ***				
**6.** Frequency of PA	0.29 ***	−0.05	−0.21 **	−0.10	−0.16 *			
**7.** LPA	0.12	−0.11	−0.24 **	−0.01	−0.10	0.12		
**8.** MVPA	0.23 **	0.05	−0.20 **	−0.06	−0.17 *	0.07	0.18 *	
Mean	3.4	4.5	7.8	6.9	69.7	2.2	346.8	38.6
Standard Devition	2.4	1.5	2.9	1.6	16.4	1.9	82.1	26.5
Skewness	0.7	−0.8	−0.2	−0.4	−0.2	1.7	0.4	1.7
Kurtosis	−0.1	−0.3	−0.7	0.3	−0.6	5.0	−0.1	4.7

* *p* < 0.05, ** *p* < 0.01, *** *p* < 0.001; PA: Physical Activity; LPA: Light PA; MVPA: Moderate-Vigorous PA.

**Table 2:** Fitted models with robust estimations, test statistics, mediation effects and path coefficients in structural models.

Models and Fit	Predictor	DV	*B*	SE	*z*		Effect Size
First Antecedents: PA self-efficacy Covariant: Fibromyalgia impact χ^2^ = 12.468(5), *p* = 0.029 CFI = 0.919 RMSEA = 0.088 90% CI (0.026–0.151) SRMR = 0.051	PA self-efficacy	LPA	−0.001	0.096	−0.011	ns	0.057
	Pain avoidance goals	LPA	−0.077	0.078	−0.987	ns	
	Activity avoidance	LPA	−0.206	0.086	−2.409	*	
	PA self-efficacy	Pain avoidance goals	−0.264	0.085	−3.100	**	0.066
	PA self-efficacy	MVPA	0.205	0.071	2.881	**	0.065
	Fibromyalgia impact	MVPA	−0.118	0.072	−1.640	ns	
	Pain avoidance goals	Activity avoidance	0.145	0.072	2.012	*	0.239
	PA self-efficacy	Activity avoidance	−0.446	0.072	−6.232	***	
Final Antecedents: PA self-efficacy χ^2^ = 5.926(4), *p* = 0.205 CFI = 0.973 RMSEA = 0.055 90% CI (0.000–0.139) SRMR = 0.041	PA self-efficacy	MVPA	0.230	0.066	3.471	**	0.050
	Activity avoidance	LPA	−0.223	0.075	−2.980	**	0.051
	PA self-efficacy	Activity avoidance	−0.446	0.071	−6.280	***	0.239
	Pain avoidance goals	Activity avoidance	0.145	0.072	2.011	*	
	PA self-efficacy	Pain avoidance goals	−0.264	0.085	−3.100	**	0.066
Serial multiple mediation effects							
Indirect_1:	PA self-efficacy → Pain avoidance goals	Pain avoidance goals → Activity avoidance	−0.038	0.022	−1.704	+	
Indirect_2:	PA self-efficacy → Activity avoidance	Activity avoidance → LPA	0.099	0.035	2.833	**	
Indirect_3	PA self-efficacy → Pain avoidance goals → Activity avoidance → LPA		0.009	0.006	1.484	ns	
Total:	Total effect of PA self-efficacy on activity avoidance		−0.484	0.068	−7.133	***	
	Total effect on LPA		−0.437	0.073	−5.989	***	

LPA: Light physical activity; MVPA: Moderate-vigorous physical activity; DV: Dependent variable; SE = Standard error; ns: non-significant; CFI: Comparative fit index; RMSEA: Root Mean Square Error of Approximation; SRMR: Standardized Root Mean Square Residual; + *p* < 0.10; * *p* < 0.05; ** *p* < 0.01; *** *p* < 0.001.

## Data Availability

The data base supporting the results is available by request to Fermin Martinez-Zaragoza (f.martinez@umh.es).

## References

[1] Karsdorp P.A., Geenen R., Kroese F.M., Vlaeyen J.W.S. (2016). Turning Pain Into Cues for Goal-Directed Behavior: Implementation Intentions Reduce Escape-Avoidance Behavior on a Painful Task. J. Pain.

[2] García-Hermoso A., Saavedra J.M., Escalante Y. (2015). Effects of exercise on functional aerobic capacity in adults with fibromyalgia syndrome: A systematic review of randomized controlled trials. J. Back Musculoskelet. Rehabil..

[3] Booth J., Moseley G.L., Schiltenwolf M., Cashin A., Davies M., Hübscher M. (2017). Exercise for chronic musculoskeletal pain: A biopsychosocial approach. Musculoskelet. Care.

[4] Crombez G., Eccleston C., Van Damme S., Vlaeyen J.W.S., Karoly P. (2012). Fear-Avoidance Model of Chronic Pain. Clin. J. Pain.

[5] Meulders A. (2019). From fear of movement-related pain and avoidance to chronic pain disability: A state-of-the-art review. Curr. Opin. Behav. Sci..

[6] Tabor A., Van Ryckeghem D.M.L., Hasenbring M.I. (2020). Pain Unstuck: The Role of Action and Motivation. Clin. J. Pain.

[7] Van Damme S., Crombez G., Eccleston C. (2008). Coping with pain: A motivational perspective. Pain.

[8] Van Damme S., Kindermans H.P.J. (2015). A self-regulation perspective on avoidance and persistence behavior in chronic pain: New theories, new challenges?. Clin. J. Pain.

[9] Arnold L.M., Choy E., Clauw D.J., Goldenberg D.L., Harris R.E., Helfenstein M., Jensen T.S., Noguchi K., Silverman S.L., Ushida T. (2016). Fibromyalgia and chronic pain syndromes: A white paper detailing current challenges in the field. Clin. J. Pain.

[10] Sarzi-Puttini P., Giorgi V., Marotto D., Atzeni F. (2020). Fibromyalgia: An update on clinical characteristics, aetiopathogenesis and treatment. Nat. Rev. Rheumatol..

[11] Häuser W., Ablin J., Fitzcharles M.A., Littlejohn G., Luciano J.V., Usui C., Walitt B. (2015). Fibromyalgia. Nat. Rev. Dis. Prim..

[12] McLoughlin M.J., Colbert L.H., Stegner A.J., Cook D.B. (2011). Are women with fibromyalgia less physically active than healthy women?. Med. Sci. Sports Exerc..

[13] Ruiz J.R., Segura-Jiménez V., Ortega F.B., Álvarez-Gallardo I.C., Camiletti-Moirón D., Aparicio V.A., Carbonell-Baeza A., Femia P., Munguía-Izquierdo D., Delgado-Fernández M. (2013). Objectively measured sedentary time and physical activity in women with fibromyalgia: A cross-sectional study. BMJ Open.

[14] Segura-Jiménez V., Borges-Cosic M., Soriano-Maldonado A., Estévez-López F., Álvarez-Gallardo I.C., Herrador-Colmenero M., Delgado-Fernández M., Ruiz J.R. (2017). Association of sedentary time and physical activity with pain, fatigue, and impact of fibromyalgia: The al-Ándalus study. Scand. J. Med. Sci. Sport.

[15] Estévez-López F., Álvarez-Gallardo I.C., Segura-Jiménez V., Soriano-Maldonado A., Borges-Cosic M., Pulido-Martos M., Aparicio V.A., Carbonell-Baeza A., Delgado-Fernández M., Geenen R. (2018). The discordance between subjectively and objectively measured physical function in women with fibromyalgia: Association with catastrophizing and self-efficacy cognitions. The al-Ándalus project. Disabil. Rehabil..

[16] Macfarlane G.J., Kronisch C., Deanv L.E., Atzeni F., Häuser W., Flub E., Choy E., Kosek E., Amris K., Branco J. (2017). EULAR revised recommendations for the management of fibromyalgia. Ann. Rheum. Dis..

[17] Andrade A., Dominski F.H., Sieczkowska S.M. (2020). What we already know about the effects of exercise in patients with fibromyalgia: An umbrella review. Semin. Arthritis Rheum..

[18] Bidonde J., Busch A.J., Schachter C.L., Webber S.C., Musselman K.E., Overend T.J., Góes S.M., Dal Bello-Haas V., Boden C. (2019). Mixed exercise training for adults with fibromyalgia. Cochrane Database Syst. Rev..

[19] Izquierdo-Alventosa R., Inglés M., Cortés-Amador S., Gimeno-Mallench L., Chirivella-Garrido J., Kropotov J., Serra-Añó P. (2020). Low-intensity physical exercise improves pain catastrophizing and other psychological and physical aspects in women with fibromyalgia: A randomized controlled trial. Int. J. Environ. Res. Public Health.

[20] Loftus N., Dobbin N., Crampton J.S. (2021). The effects of a group exercise and education programme on symptoms and physical fitness in patients with fibromyalgia: A prospective observational cohort study. Disabil. Rehabil..

[21] O’Connor S.R., Tully M.A., Ryan B., Bleakley C.M., Baxter G.D., Bradley J.M., McDonough S.M. (2015). Walking exercise for chronic musculoskeletal pain: Systematic review and meta-analysis. Arch. Phys. Med. Rehabil..

[22] O’Dwyer T., Maguire S., Mockler D., Durcan L., Wilson F. (2019). Behaviour change interventions targeting physical activity in adults with fibromyalgia: A systematic review. Rheumatol. Int..

[23] Pulido-Martos M., Luque-Reca O., Segura-Jiménez V., Álvarez-Gallardo I.C., Soriano-Maldonado A., Acosta-Manzano P., Gavilán-Carrera B., McVeigh J.G., Geenen R., Delgado-Fernández M. (2020). Physical and psychological paths toward less severe fibromyalgia: A structural equation model. Ann. Phys. Rehabil. Med..

[24] Steiner J.L., Bigatti S.M., Ang D.C. (2015). Trajectory of change in pain depression and physical functioning after physical activity adoption in fibromyalgia. J. Health Psychol..

[25] Bidonde J., Boden C., Foulds H., Kim S.Y., Ablin J.N., Shoenfeld Y. (2021). Physical Activity and Exercise Training for Adults with Fibromyalgia. Fibromyalgia Syndrome.

[26] Sanz-Baños Y., Pastor-Mira M.-Á., Lledó A., López-Roig S., Peñacoba C., Sánchez-Meca J. (2018). Do women with fibromyalgia adhere to walking for exercise programs to improve their health? Systematic review and meta-analysis. Disabil. Rehabil..

[27] Pastor-Mira M.-A., López-Roig S., Martínez-Zaragoza F., León E., Abad E., Lledó A., Peñacoba C. (2019). Goal Preferences, affect, activity patterns and health outcomes in women with fibromyalgia. Front. Psychol..

[28] Kindermans H.P.J., Roelofs J., Goossens M.E.J.B., Huijnen I.P.J., Verbunt J.A., Vlaeyen J.W.S. (2011). Activity patterns in chronic pain: Underlying dimensions and associations with disability and depressed mood. J. Pain.

[29] Racine M., Galán S., De La Vega R., Pires C.T., Solé E., Nielson W.R., Miró J., Moulin D.E., Jensen M.P. (2018). Pain-related Activity Management Patterns and Function in Patients with Fibromyalgia Syndrome. Clin. J. Pain.

[30] López-Roig S., Pastor M.A., Peñacoba C., Lledó A., Sanz Y., Velasco L. (2016). Prevalence and predictors of unsupervised walking and physical activity in a community population of women with fibromyalgia. Rheumatol. Int..

[31] Bandura A. (1986). Social Foundations of Thought and Action: A Social Cognitive Theory.

[32] Bandura A. (1997). Self-Efficacy: The Exercise of Control.

[33] Young M.D., Plotnikoff R.C., Collins C.E., Callister R., Morgan P.J. (2014). Social cognitive theory and physical activity: A systematic review and meta-analysis. Obes. Rev..

[34] Collado-Mateo D., Lavín-Pérez A.M., Peñacoba C., Del Coso J., Leyton-Román M., Luque-Casado A., Gasque P., Fernández-Del-olmo M.Á., Amado-Alonso D. (2021). Key factors associated with adherence to physical exercise in patients with chronic diseases and older adults: An umbrella review. Int. J. Environ. Res. Public Health.

[35] Rhodes R.E., Boudreau P., Josefsson K.W., Ivarsson A. (2021). Mediators of physical activity behaviour change interventions among adults: A systematic review and meta-analysis. Health Psychol. Rev..

[36] Williams S.L., French D.P. (2011). What are the most effective intervention techniques for changing physical activity self-efficacy and physical activity behaviour—And are they the same?. Health Educ. Res..

[37] Jackson T., Wang Y., Wang Y., Fan H. (2014). Self-efficacy and chronic pain outcomes: A meta-analytic review. J. Pain.

[38] Lee H., Hübscher M., Moseley G.L., Kamper S.J., Traeger A.C., Mansell G., McAuley J.H. (2015). How does pain lead to disability? A systematic review and meta-analysis of mediation studies in people with back and neck pain. Pain.

[39] Kaleth A.S., Bigatti S.M., Slaven J.E., Kelly N., Ang D.C. (2020). Predictors of physical activity in patients with fibromyalgia. J. Clin. Rheumatol..

[40] Oliver K., Cronan T.A. (2005). Correlates of physical activity among women with fibromyalgia syndrome. Ann. Behav. Med..

[41] Wolfe F., Smythe H.A., Yunus M.B., Bennett R.M., Bombardier C., Goldenberg D.L., Tugwell P., Campbell S.M., Abeles M., Clark P. (1990). The american college of rheumatology 1990 criteria for the classification of fibromyalgia. Arthritis Rheum..

[42] Wolfe F., Clauw D.J., Fitzcharles M.A., Goldenberg D.L., Häuser W., Katz R.L., Mease P.J., Russell A.S., Russell I.J., Walitt B. (2016). Revisions to the 2010/2011 fibromyalgia diagnostic criteria. Semin. Arthritis Rheum..

[43] López-Roig S., Pastor-Mira M.-Á., Núñez R., Nardi A., Ivorra S., León E., Peñacoba C. (2021). Assessing Self-Efficacy for physical activity and walking exercise in women with fibromyalgia. Pain Manag. Nurs..

[44] Pastor M.Á., López-Roig S., Sanz Y., Peñacoba C., Cigarán M., Velasco L., Écija C. (2015). Walking as physical exercise in Fibromyalgia: An elicitation study from the Theory of Planned Behavior. An. Psicol..

[45] Karsdorp P.A., Vlaeyen J.W.S. (2011). Goals matter: Both achievement and pain-avoidance goals are associated with pain severity and disability in patients with low back and upper extremity pain. Pain.

[46] Pastor M.-Á., López-Roig S., Lledó A., Peñacoba C., Velasco L., Schweiger-Gallo I., Cigarán M., Écija C., Limón R., Sanz Y. (2014). Combining motivational and volitional strategies to promote unsupervised walking in patients with fibromyalgia: Study protocol for a randomized controlled trial. Trials.

[47] Munguía-Izquierdo D., Legaz-Arrese A., Mannerkorpi K. (2011). Transcultural adaptation and psychometric properties of a Spanish-language version of physical activity instruments for patients with fibromyalgia. Arch. Phys. Med. Rehabil..

[48] Esteve R., Ramírez-Maestre C., Peters M.L., Serrano-Ibáñez E.R., Ruíz-Párraga G.T., López-Martínez A.E. (2016). Development and initial validation of the activity patterns scale in patients with chronic pain. J. Pain.

[49] López-Roig S., Peñacoba C., Martínez-Zaragoza F., Abad E., Catalá P., Suso-Ribera C., Pastor-Mira M.A. (2021). The Activity Patterns Scale: An analysis of its construct validity in women with fibromyalgia. Clin. J. Pain.

[50] Salgueiro M., García-Leiva J.M., Ballesteros J., Hidalgo J., Molina R., Calandre E.P. (2013). Validation of a Spanish version of the Revised Fibromyalgia Impact Questionnaire (FIQR). Health Qual. Life Outcomes.

[51] Aguilar-Farías N., Brown W.J., Peeters G.M.E.E.G. (2014). ActiGraph GT3X+ cut-points for identifying sedentary behaviour in older adults in free-living environments. J. Sci. Med. Sport.

[52] Muthén L.K., Muthén B.O. (2002). How to Use a Monte Carlo Study to Decide on Sample Size and Determine Power. Struct. Equ. Model..

[53] R Core Team (2020). R: A Language and Environment for Statistical Computing. http://www.r-project.org/.

[54] Rosseel Y. (2012). Iavaan: An R Package for Structural Equation Modeling. J. Stat. Softw..

[55] Raykov T., Tomer A., Nesselroade J. (1991). Reporting structural equation modeling results in Psychology. Psychol. Aging.

[56] Korkmaz S., Goksuluk D., Zararsiz G. (2014). M.V.N.: An R Package for Assessing Multivariate Normality. R J..

[57] Hu L., Bentler P.M. (1999). Cutoff criteria for fit indexes in covariance structure analysis: Conventional criteria versus new alternatives. Struct. Equ. Model. Multidiscip. J..

[58] Cohen J. (1988). Statistical Power Analysis for the Behavioral Sciences.

[59] Lin I., Wiles L., Waller R., Goucke R., Nagree Y., Gibberd M., Straker L., Maher C.G., O’Sullivan P.P.B. (2020). What does best practice care for musculoskeletal pain look like? Eleven consistent recommendations from high-quality clinical practice guidelines: Systematic review. Br. J. Sports Med..

[60] Catalá P., Lopez-Roig S., Ecija C., Suso-Ribera C., Peñacoba C. (2021). Why do some people with severe chronic pain adhere to walking prescriptions whilst others won’t? A cross-sectional study exploring clinical and psychosocial predictors in women with fibromyalgia. Rheumatol. Int..

[61] Perruchoud C., Buchser E., Johanek L.M., Aminian K., Paraschiv-Ionescu A., Taylor R.S. (2014). Assessment of physical activity of patients with chronic pain. Neuromodulation.

[62] McEwan D., Harden S.M., Zumbo B.D., Sylvester B.D., Kaulius M., Ruissen G.R., Dowd A.J., Beauchamp M.R. (2016). The effectiveness of multi-component goal setting interventions for changing physical activity behaviour: A systematic review and meta-analysis. Health Psychol. Rev..

[63] Andrews N.E., Strong J., Meredith P.J. (2012). Activity pacing, avoidance, endurance, and associations with patient functioning in chronic pain: A systematic review and meta-analysis. Arch. Phys. Med. Rehabil..

[64] Gourlan M., Bernard P., Bortolon C., Romain A.J., Lareyre O., Carayol M., Ninot G., Boiché J. (2016). Efficacy of theory-based interventions to promote physical activity. A meta-analysis of randomised controlled trials. Health Psychol. Rev..

